# Emotional awareness and expression therapy vs cognitive behavioural therapy in patients with chronic pain: Systematic review and Meta Analysis

**DOI:** 10.1192/j.eurpsy.2025.376

**Published:** 2025-08-26

**Authors:** N. Farid, S. Reza, K. Marvin, R. Sabloak, N. Belhayne, R. Jabbar, I. Teohar

**Affiliations:** 1 Medicine, Mohiuddin Islamic medical College, Mirpur, Pakistan; 2 Upszilla health complax, Sitakundo, Bangladesh; 3 Azerbaijan Medical University, Baku, Azerbaijan; 4 Faculty of medicine, Sriwijaya University, Palembang, Indonesia; 5 Faculty of rehabilitationsciences and health techniques, Universiapolis University, Agadir, Morocco; 6 Fatima Memorial Hospital, FMHCMD, Lahore, Pakistan; 7 Department of Physical and Rehabilitation Medicine, The Clinical Rehabilitation Hospital of Cluj-Napoca, Cluj Napoca, Romania

## Abstract

**Introduction:**

Emotional awareness and expression therapy (EAET) is a newer approach that focuses on identifying and expressing repressed emotions.While cognitive behavioural therapy (CBT) has ample evidence supporting its efficacy, the benefits provided by EAET are still unknown.

**Objectives:**

We aimed to compare the efficacy of EAET versus CBT in treating chronic pain and stress-related conditions.

**Methods:**

We systematically searched PubMed, Embase, Cochrane and Web of Science databases for randomized controlled trials (RCTs) comparing EAET with CBT in patients with chronic pain. Statistical analysis was performed using Review Manager 8.1.1 (Cochrane Collaboration). Heterogeneity was assessed by I². We pooled mean differences (MD) with 95% confidence intervals (CI). Reduction in pain severity was assessed using brief pain inventory (BPI), anxiety by PROMIS anxiety short form 7a, sleep disturbances by PROMIS sleep disturbances short form 8a and satisfaction with life by NIH toolbox general life satisfaction fixed form B.

**Results:**

Three RCTs reporting data on 333 patients were included.Among them, 173 (52%) received EAET and 160 (48%) received CBT. Follow-up ranged from 3 to 6 months. The mean age of patients between studies ranged from 48 to 75 years. EAET significantly reduced pain severity (MD -0.93 points; 95% CI -1.63 to -0.23 ; p=0.009; I² = 81%) compared with CBT. There were no differences in anxiety (MD -1.62 points; 95% CI -4.30 to 1.05; p=0.23; I² = 91%), Sleep disturbance (MD -0.21 points; 95% CI -0.55 to 0.12; p=0.22; I² = 55%) and satisfaction with life (MD 0.71 points; 95% CI -0.24 to 1.65; p=0.14; I²=94%).

**Conclusions:**

In patients with chronic pain, EAET was associated with a greater reduction in pain severity compared with CBT.

**Disclosure of Interest:**

None Declared

